# In Vitro Shear Stress Measurements Using Particle Image Velocimetry in a Family of Carotid Artery Models: Effect of Stenosis Severity, Plaque Eccentricity, and Ulceration

**DOI:** 10.1371/journal.pone.0098209

**Published:** 2014-07-09

**Authors:** Sarah Kefayati, Jaques S. Milner, David W. Holdsworth, Tamie L. Poepping

**Affiliations:** 1 Department of Physics and Astronomy, University of Western Ontario, London, ON, Canada; 2 Imaging Research Laboratories, Robarts Research Institute, London, ON, Canada; 3 Department of Surgery, University of Western Ontario, London, ON, Canada; University of California Berkeley, United States of America

## Abstract

Atherosclerotic disease, and the subsequent complications of thrombosis and plaque rupture, has been associated with local shear stress. In the diseased carotid artery, local variations in shear stress are induced by various geometrical features of the stenotic plaque. Greater stenosis severity, plaque eccentricity (symmetry) and plaque ulceration have been associated with increased risk of cerebrovascular events based on clinical trial studies. Using particle image velocimetry, the levels and patterns of shear stress (derived from both laminar and turbulent phases) were studied for a family of eight matched-geometry models incorporating independently varied plaque features – i.e. stenosis severity up to 70%, one of two forms of plaque eccentricity, and the presence of plaque ulceration). The level of laminar (ensemble-averaged) shear stress increased with increasing stenosis severity resulting in 2–16 Pa for free shear stress (FSS) and approximately double (4–36 Pa) for wall shear stress (WSS). Independent of stenosis severity, marked differences were found in the distribution and extent of shear stress between the concentric and eccentric plaque formations. The maximum WSS, found at the apex of the stenosis, decayed significantly steeper along the outer wall of an eccentric model compared to the concentric counterpart, with a 70% eccentric stenosis having 249% steeper decay coinciding with the large outer-wall recirculation zone. The presence of ulceration (in a 50% eccentric plaque) resulted in both elevated FSS and WSS levels that were sustained longer (∼20 ms) through the systolic phase compared to the non-ulcerated counterpart model, among other notable differences. Reynolds (turbulent) shear stress, elevated around the point of distal jet detachment, became prominent during the systolic deceleration phase and was widely distributed over the large recirculation zone in the eccentric stenoses.

## Introduction

The association between shear stress and atherosclerosis-related complications is well established [1], [2]. Early stages of atherogenesis and plaque localization in the carotid artery are associated with regions of low or highly oscillatory wall shear stress (WSS), while initially the regions with moderate to high shear stress are protected. Once stenotic plaque has formed and matured, shear stress forces – now altered due to the changed vessel geometry – play a role in plaque instability [3], [4]. With regard to plaque rupture and flow-induced shear stress forces, conflicting results have been reported. For instance, Groen et al. [5] reported that the site of plaque rupture coincided with the region of elevated WSS. In contrast, Leach et al. [6] showed that the region of plaque rupture is in fact associated with a local minimum WSS. In addition to flow-induced WSS, several studies [7], [8] have shown the possible association between plaque rupture and increased biomechanical structural stresses on the plaque. For example, Teng et al. [9] suggested that plaque maximum wall stress (i.e. maximum principal stress) is a better indicator of plaque rupture than maximum WSS at the site of plaque. The study of high WSS, as well as high spatial WSS gradient, has gained much attention due to its known association not only with plaque destabilization and thrombosis in stenosed arteries, but also with outward remodeling of vessels and development of saccular intracranial aneurysms [10].

Atherosclerotic plaques are particularly predisposed to thrombogenicity, which also can be promoted further by pathological shear stresses. Thrombosis is a complicated multi-step process that, under certain conditions, can occur either at the site of plaque or the vicinity of the vessel wall. Since the late 1970s, numerous investigations have been conducted, mainly under controlled conditions, to identify the underlying mechanism of shear-induced thrombus formation and mobilization. In an intriguing study by Nesbitt et al. [11], it was found that pathological WSS at the site of stenosis spatially regulates thrombus growth; when a shear-acceleration zone is followed by a shear-deceleration zone (labelled as a shear microgradient), platelet aggregation forms at the post-stenotic expansion zone starting from the stenosis apex. This mechanism of aggregation – in which soluble agonists merely play a secondary role – is rheology dependent with platelets only at a low level of activation. The intrinsic instability of these post-stenotic aggregates can contribute to thromboembolism and consequent cerebrovascular events [12].

For moderate and severe stenosis, turbulent (Reynolds) shear stress is expected distal to the stenosis throat where the jet flow becomes disturbed and turbulent. While RSS does not directly relate to a force seen by the platelets in the same way as laminar shear stress, the significance of Reynolds shear stresses should not be dismissed. Reynolds stresses and blood cell damage are well correlated [13] and thus play a significant role in the prediction of mechanical hemolysis. In fact, RSS may reflect *effective* shear stresses due to viscous cell-cell interactions during the dissipation of turbulent eddies as proposed by Antiga and Steinman [14] who showed that, under turbulent conditions and considering the high concentration of red blood cells in vivo, viscous cell-cell interactions may give rise to increased shear-stress forces on the same order as Reynolds shear stresses.

An often-cited threshold of 400 Pa [13] is considered sufficient for lysis of red blood cells (and arguably considered to be an underestimation [15]). Platelets, however, are much more sensitive to shear, and their deformation starts at shear stress levels as low as 10–16.5 Pa [16]. It also has been established that shear-induced platelet activation has a cumulative effect, meaning it depends not only on the shear level, but also on the duration of exposure time [17]–[19]; at higher shear-stress levels, shorter exposure time is required. Turbulent stress levels of 10–100 Pa are considered to trigger platelet activation [20].

Through flow alteration, the dynamics of shear stress are influenced by local geometrical features of the plaque. Greater stenosis severity, plaque eccentricity (symmetry) and plaque ulceration are among the plaque features that have been associated with more frequent cerebrovascular events based on clinical trial studies [21]–[23]. From a hemodynamic perspective, this may be due to an increased level of shear stress, such as seen with more severe stenosis, resulting in an increased probability of plaque rupture and larger formation of unstable thrombi formed at the post-stenotic region. However, independent of stenosis severity, plaque eccentricity also can impact the local hemodynamics of the carotid artery. In a study conducted in two matched models of mild stenosis with symmetric and asymmetric form, Kaazempur-Mofrad et al. [24] showed that stenosis asymmetry is associated with the development of secondary flows that effect the mass transfer. In a previous computational simulation study by Tambasco and Steinman [25], path-dependent hemodynamic factors associated with thrombotic activity were investigated in 30% concentrically and eccentrically stenosed models of the carotid bifurcation with identical geometries as used in the present work. Using the trajectory scheme, the dynamic path histories of simulated blood elements (e.g. platelets) were derived from which the coupled effect of particle transit time and shear stress was obtained. Compared to the concentric model, the eccentric counterpart was found to be suggestive of a higher level of thrombotic activity owing to the larger percentage of particles experiencing maximum shear-stress exposure, threshold activation, and mural deposition.

The widely recognized significance of hemodynamic factors of the carotid artery bifurcation with respect to stroke risk has encouraged numerous studies, mainly performed using computational techniques [26]–[28], with fewer *in vitro* studies [29], [30]. Regardless of the applied technique, the majority of these investigations have been conducted each in a different patient-specific or idealized model of the carotid artery, and only a few computational fluid dynamics (CFD) studies have reported the effects due to changes in geometrical features of the stenotic plaques [25], [31], [32]. Assessment of the individual effect of each geometrical feature by performing a pooled comparison across these studies is difficult due to variation in geometries, modalities, and flow conditions.

The objective of the present work was to conduct a comprehensive matched-model study to assess the shear stress (both laminar and turbulent) affected by three independent geometrical stenosis features (stenosis severity, plaque eccentricity, and plaque ulceration) that are each associated with increased cerebrovascular events. Flow was characterized experimentally using particle image velocimetry (PIV) in a set of physiologically relevant carotid artery models covering a wide range of disease progression.

## Methods

### Experimental set-up

**Figure 1:**
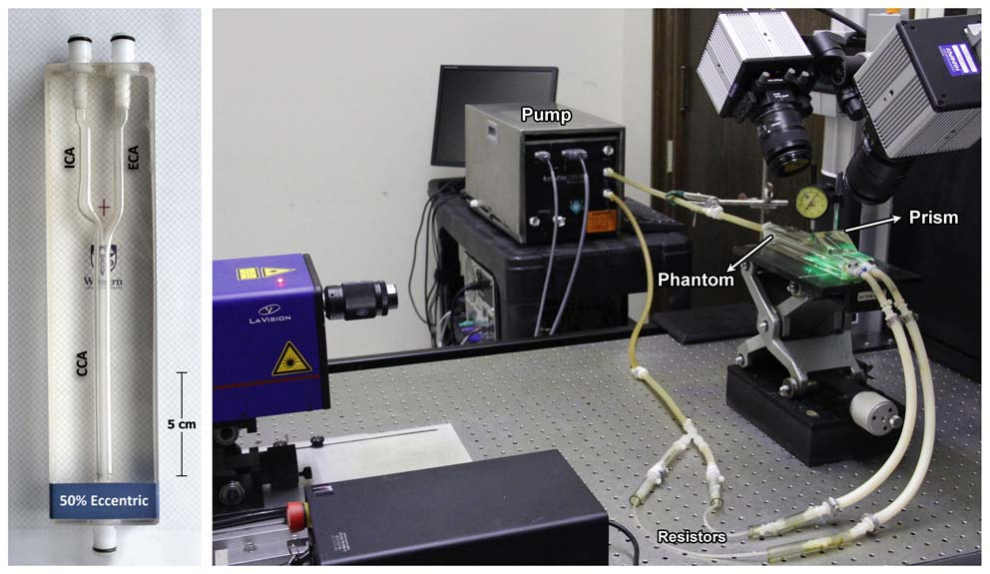
Example of one of the carotid-artery bifurcation phantoms incorporating a 50% eccentric stenosis (a), and a picture of the experimental set-up depicting the flow circuit and PIV components (b).

**Figure 2:**
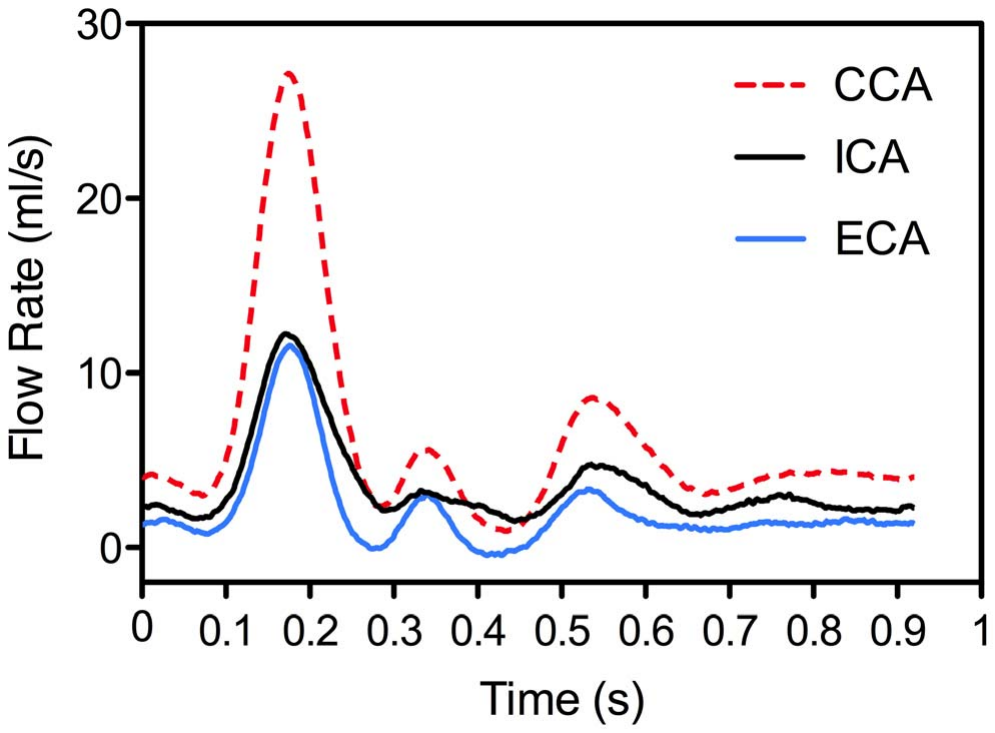
Volumetric flow-rate waveforms, measured using electromagnetic flow meters, at the inlet of the CCA and outlets of the ICA and ECA.

A family of eight carotid artery phantoms was examined consisting of six models with 30%, 50%, and 70% stenosis severity (based on NASCET criteria) each in either form of the extreme plaque symmetry (concentric or eccentric), as well as a 50% eccentrically stenosed plaque with an ulceration incorporated and a normal (disease-free) model. The PIV-compatible life-sized phantoms [33] each incorporated the physiologically relevant geometry [34] based on an 8-mm inner diameter common carotid artery (CCA), 5.5-mm internal carotid artery (ICA), and 4.5-mm external carotid artery (ECA). The flow loop included a phantom, downstream flow resistors (generating ∼60∶40 ICA∶ECA flow division), and a programmable positive-displacement pump (CompuFlow 1000, Shelley Medical Imaging, London CAN) [35] equipped with synchronization signal thus enabling gated data acquisition. Figure 1 shows the experimental set-up including one of the carotid phantoms. The pump was programmed with an idealized carotid waveform [36] with a maximum flow rate at peak systole of 27.13 (±0.30) mL/s and a mean of 6.29 (±0.09) mL/s, as measured at the inlet of the CCA. Figure 2 shows the measured flow-rate waveforms at the CCA inlet and the ICA and ECA outlets. Local maximum and mean Reynolds numbers derived from the narrowest diameter of the ICA stenosis were 1162 and 312 in the 30% models, 1627 and 473 in the 50% models, and 2712 and 789 in the 70% stenosed models. The phantoms were perfused with a blood-mimicking fluid [37], specifically developed to match the refractive index (n = 1.4140±0.0008) of the silicone PIV phantoms for reduced optical distortion and with corresponding dynamic viscosity (*μ* = 4.31±0.03 mPa s) within the average range of healthy human blood (4.4±0.5 mPa s) as cited by Yousif et al, which also aligns with the viscosity of standard ultrasound blood-mimicking fluid [38]. The fluid was seeded with polymer fluorescent microspheres with 15-*µm* mean diameter (FLUOSTAR, EBM Corp., Tokyo, Japan). The density of the particles was in close proximity to the density of the blood-mimicking fluid (*ρ* = 1.244 g/ml).

Velocity maps were obtained using a stereoscopic PIV system (LaVision Inc., Ipsilanti MI, USA), as previously described in detail [39] and summarized in Table 1. Briefly, the system consisted of a 5.5-W, 532-nm Nd∶YAG laser creating a 1.5-mm thick horizontal illumination sheet through the phantom, which was viewed stereoscopically from above using CMOS cameras fitted with Scheimpflug adapters and 550-nm filters suitable for the fluorescent microspheres. An overlying silicone prism was used to reduce astigmatism and was placed on top of the silicone phantom symmetrically between the two cameras with an oblique viewing angle of ∼35°.

**Table 1:** PIV data acquisition and analysis parameters.

Laser & illumination	5.5 W, 532-nm continuous wave Nd∶YAG,
	Time interval: min. 110 µs (70% stenosed), max. 400 µs (normal model)
	Illumination width: min. 100 µs
Camera & acquisition	1024×1024 pixels, 10-bit CMOS
	Sensor pitch size: 17 µm
	Acquisition rate: 1 kHz (500 Hz double frame)
Image properties	Viewing angle: ±35°
	Lens focal length: 60 mm
	Lens focal aperture: 2.8
	Filters: 550-nm long-pass
	Field of view: ∼45×41 mm^2^
	Magnification: ∼38 µm per pixel
Image pre-processing	Sliding background subtraction
	RMS mask to eliminate non-flow regions
Particles & image density	15-µm Rhodamine-B infused polystyrene 2.5 pixels mean
	2.5 pixels mean image particle size
	Average of ∼9 particles per 16×16 px^2^ window
Vector analysis	No. of measurement planes per model: ∼15 No. of velocity
	No. of velocity vectors per model: ∼21,000
	Type: adaptive multi-pass processing with decreasing window size from 64×64 to 16×16, using 3 refinement passes
	Final grid spacing: 8 pixels (∼0.3 mm)
Vector validation	Median filter (remove and replace)
	Single pass smoothing (3×3)

### Data acquisition and analysis

A summary of PIV data acquisition and analysis parameters is provided in Table 1. For each model, the initial 870-ms portion of the cardiac cycle was captured from the full 920-ms cycle, as limited by the on-board camera memory. For volumetric acquisitions, 15 measurement planes throughout the lumen volume were acquired with elevation increments of 0.5 mm. For each plane, double-frame images were recorded at 100 Hz (10-ms temporal resolution).

PIV data processing was performed using DaVis 7.2 (LaVision). The calibration procedure and velocity-evaluation algorithm have previously been described in detail [40]. Before the velocity processing, a root-mean-square (RMS) mask, created specifically for each plane of measurement from a full set of images, was applied on each particle image in order to eliminate the non-flow regions. The velocity-evaluation algorithm was a fast-Fourier-transform based cross-correlation analysis, which was employed in an adaptive multi-pass scheme with decreasing interrogation-window size ranging from 64×64 pixels down to 16×16 with 50% overlap throughout. The calibrated particle images had a scaling factor of ∼38 µm per pixel resulting in vector maps with a grid resolution of ∼0.30 mm. The final planar three-component (2D-3C) vector fields were post-processed once with a 3×3-smoothing kernel.

As noted in the study by Kähler et al. [41], when using a window correlation algorithm for PIV, displacements estimated within half of an interrogation window (in our case less than 8 pixels) normal to the wall are biased and overestimated. Thus, for our velocity fields, the last row of vectors located within half of the interrogation window (i.e. 8 pixels) in the wall-normal direction were eliminated and assumed to be zero (which is reasonable as the potentially overestimated velocities were already close to zero).

All further processing on the velocity maps was performed using custom MATLAB programs. Shear stress parameters were estimated for a 180-ms window corresponding to the systolic phase, starting 20 ms before peak systole and continuing into the deceleration phase of post peak-systole.

The 2D-3C velocity maps obtained from 15 repeated cardiac cycles were used to calculate an ensemble (phase)-averaged velocity field, which was subtracted from the instantaneous velocity to yield components of velocity fluctuation based on Reynolds decomposition 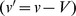 [42]. The phase-averaged flow represents the coherent (i.e. deterministic) part of the flow, and any deviation (i.e. fluctuation) from it can be associated with the turbulent (i.e. incoherent) characteristics. In the present study, the required phase correlation was facilitated through synchronized data acquisition triggered by a pump synchronization signal. At each time point (t) of the cardiac cycle, the ensemble-averaged velocity maps were used to represent laminar flow behavior applied to the calculation of laminar shear stress defined based on the strain tensor composed of averaged velocity components as follows:
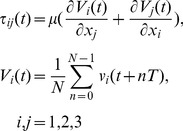
(1)where *μ* is the dynamic viscosity (4.31±0.03 cP) of the blood-mimicking fluid, *T* is the period of the cardiac cycle (920 ms), and *N* is the number of collected cardiac cycles (15). The selection of 15 cardiac cycles was based on a previous statistical analysis [40] that identified the minimum number of cycles for which turbulence intensity (standard deviation) did not differ significantly from the convergence value seen with a greater number of cycles. *V_1_*, *V_2_*, and *V_3_* correspond respectively to the ensemble (phase)-averaged velocity means (*U*, *V*, *W*) of the instantaneous velocity components *u*, *v*, and *w* in the *x*, *y*, and *z* direction (i.e. *x_1_*, *x_2_*, *x_3_*). Over the entire velocity map, including the points adjacent to the wall, velocity gradients were calculated using a central difference scheme. For the first non-zero value adjacent to the wall, the value of zero representing the wall was applied in central differencing. By assessing the line-extracted velocity profiles [40], it can be found that velocities near the wall in the ICA do not follow a linear decay (to zero) and thus, employing the forward (or backward) difference scheme for near-wall velocity gradients results in faulty values and misestimation of wall position. After velocity gradients were calculated, the shear stress tensor was solved for eigenvalues, which yields three values representing principal stresses (i.e. normal stresses in principal directions). By definition, the half difference between the maximum and minimum eigenvalue is used to represent the maximum shear stress [43]. Maximum shear stress values at the wall boundaries were then used to represent wall shear stress (WSS). For improved visualization of WSS, a smoothed-wall model and processing operations were carried out in the open-source Visualization Toolkit software (VTK version 5.10, Clifton Park, NY, USA). Shear stress values greater than zero were retained via thresholding. Extraction of the outer polygonal surface produced a coarse wall model. Linear surface subdivision was used to increase triangle vertex density and to aid in smoothing during a volume-preserving geometric smoothing step that followed. Volumetric PIV data were then resampled onto the smoothed wall model producing surface maps of the data for visualization.

The turbulent shear tress tensor, composed of Reynolds shear stress (RSS) components, was formed from the covariance of the velocity fluctuations (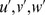) calculated using multiple repeats of the cardiac cycle (i.e. N = 15):
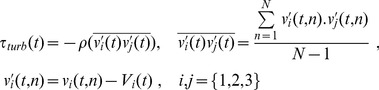
(2)where *ρ* is the density (1244 kg/m^3^) of the blood-mimicking fluid, 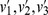 represent 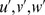 respectively, and *V_1_*, *V_2_*, *V_3_* correspond respectively to the ensemble-averaged velocity components (*U*, *V*, *W*). The normal components of the covariance tensor (i.e. velocity variances) are commonly referred to as the turbulence intensity (TI) components [40].

The total of laminar (Eqn. 1) and turbulent (Eqn. 2) shear stress were used to compose the tensor of the total shear stress (TSS), and similarly half of the difference between the largest and smallest eigenvalues was used to represent the TSS principal values.

## Results

### Laminar shear stress

**Figure 3:**
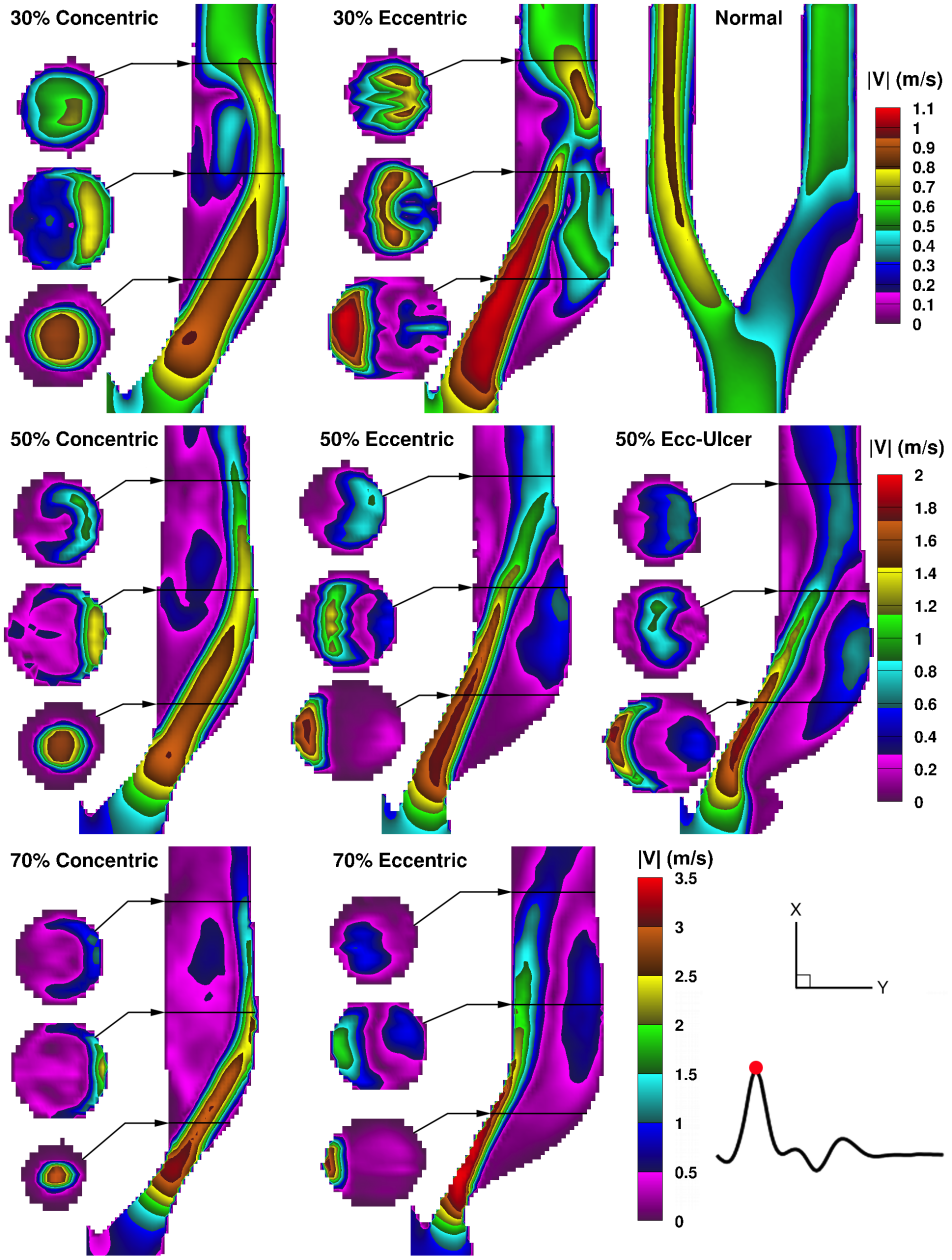
Color maps of ensemble-averaged velocity magnitudes shown for peak systole in a family of eight carotid bifurcation models. Three cross-sectional slices are shown alongside the ICA lumen, for each stenosed model, at the locations indicated by the black horizontal lines at approximately 1, 2, and 3 CCA diameters (i.e. ∼8, 16, 24 mm) downstream of the bifurcation apex. For scale, note the downstream ICA diameter is 5.5 mm. Note the range-appropriate color bar for each row of models.

Figure 3 shows the ensemble-averaged velocity magnitude for the time point of maximum flow rate (i.e. peak systole at t = 180 ms on Fig. 2) in the eight models. Additionally, central-plane velocity maps of all eight models over the course of the entire cardiac cycle are shown in synchrony in the Movie S1, and CCA velocity profiles from eight selected time points are shown in Figure S1. In the normal model, low-velocity recirculating flow occurs in the ICA bulb, while in the ECA branch, higher velocity laminar flow is seen; ECA flow was similar in all the other models. In the stenosed models, the jet follows a different path in the eccentric versus concentric models, thus creating distinct recirculation areas. In the concentric models, the Dean-type vortices seen in the second cross-sectional slices represent a helical swirling motion in the jet-free region – located distal to the stenosis throat, medial to the jet, and along the inner (flow-divider) wall – as well as reversed flow along the inner wall forming an “inner-recirculation zone”. In the eccentric models, a large “outer-recirculation zone” and a smaller inner one are created on opposite sides of the jet as it crosses over from the inner wall to the outer (non-flow-divider) wall. Also, with an eccentric stenosis, the jet enters the bifurcation at a straighter angle, and therefore due to preservation of the path trajectory, the distal jet is narrower and thus has a higher maximum velocity (by ∼10%, 3%, and 8% in the 30%, 50%, and 70% eccentrically stenosed models respectively) compared to their counterpart concentric model. Regardless of the plaque symmetry, flow relaminarizes but increasingly further downstream with increasing stenosis severity, resulting in a larger extent of the recirculation zones.

The addition of ulceration to the 50% eccentric model resulted in an even narrower jet (Fig. 3), and thus slightly faster (by ∼5%), compared to its counterpart non-ulcerated eccentric model. As a result, a slightly larger recirculation zone is created alongside the jet, extending further upstream into the stenosis throat. In the ulcer itself, very low velocities are present, which also present a risk for thrombosis, particularly when in contact with the potentially thrombogenic inner-plaque components.

**Figure 4:**
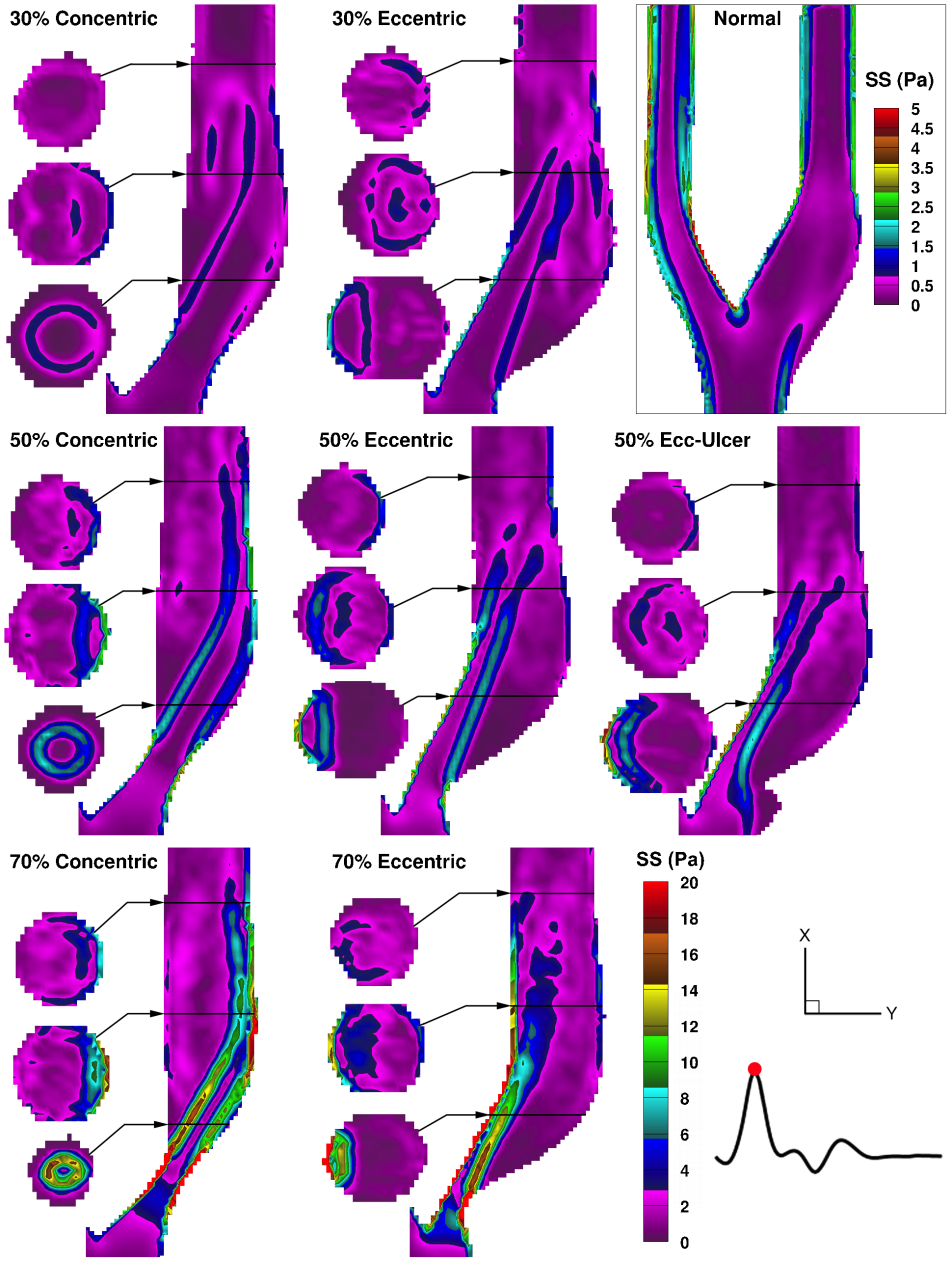
Color maps of the laminar (ensemble-averaged) shear stress shown for peak systole in the eight models. Three cross-sectional slices are shown alongside the ICA lumen for the locations indicated (as described in Fig. 3). Note the bottom right color bar represents the seven stenosed models, and a separate color bar is used for the normal model as indicated in the enclosed box.

Laminar shear stress profiles are shown in Fig. 4 for the time point of peak systole. Intraluminal laminar shear stress, herein referred to as free shear stress (FSS) (i.e. not wall related, not to be confused with FSS, flow shear stress, in the literature [9]), is governed by the pattern of the high-velocity jet. In the concentric models, immediately distal to the stenosis throat, a cylindrical lamina of FSS is evident in the lower cross-sectional slice, which can also be seen in the central-plane map as two parallel elevated-FSS streams. In the eccentric models, the shear-stress laminae are bounded on one side by the inner wall, leading to an extended region of elevated WSS along the inner wall, parallel to an extended elevated-FSS layer alongside the large outer-wall recirculation zone.

In the ulcerated model, the FSS layer starts further upstream compared to the non-ulcerated counterpart, forming ∼2 mm upstream of the stenosis throat at the mouth of the ulceration, due to the low circulating flow in the ulcer crater adjacent to the high-velocity jet.

**Figure 5:**
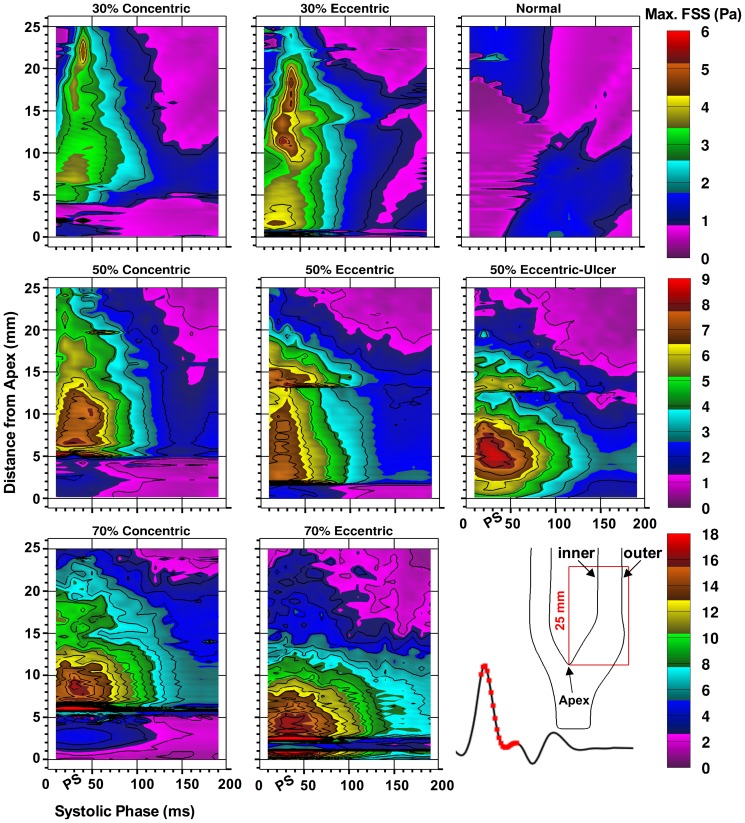
Spatial and temporal distribution of the maximum free (i.e. non wall) shear stress in the eight models shown for a 180-ms window, incorporating peak systole (PS), as indicated on the reference flow-rate waveform at the bottom right. For each time point, maximum FSS values were extracted across the entire ICA lumen along 25(0 mm). Note the range-appropriate color bar associated with each row of models and the 1-Pa isocontour increments used for all maps.

To assess the spatial and temporal distribution of the FSS, maximum FSS values were extracted over the systolic phase (corresponding to t = 160–340 ms in Fig. 2) in the ICA central plane extending 25 mm distal to the bifurcation apex, as shown in Fig. 5. The apparent spatial discontinuities mark the detachment of the jet from the wall, with elevated WSS thus converting to elevated FSS, and vice versa. In the normal (disease-free) model, maximum FSS just exceeds 2 Pa. Prior to peak systole, the maximum FSS values appear outside the bulb in the straight part of ICA lumen; however, post peak systole, the bulb area exhibits a higher level of maximum FSS due to the instability of the recirculation zone in the bulb. In all of the examined stenosed models, the maximum FSS peaks around peak systole. Overall, the maximum-FSS ranges were found to be similar in each pair of models with equal stenosis severity, reaching a peak value of 4–5 Pa in the 30% models, 7–8 Pa in 50% models (non-ulcerated), and 16–17 Pa in the 70% models. In each eccentric model, compared to the matched concentric one, the FSS layer extends shorter distally, but also forms more proximal to the stenosis (Fig. 4) resulting in similar extents of the FSS layer. This is due to the fact that the detachment of the jet from the plaque wall occurs more distally in the concentric models. The ulcerated model shows greater temporal extension later into the cardiac cycle by about 20 ms (e.g. compare 3-Pa contour level) and a higher overall peak value (9 Pa compared to 7–8 Pa).

**Figure 6:**
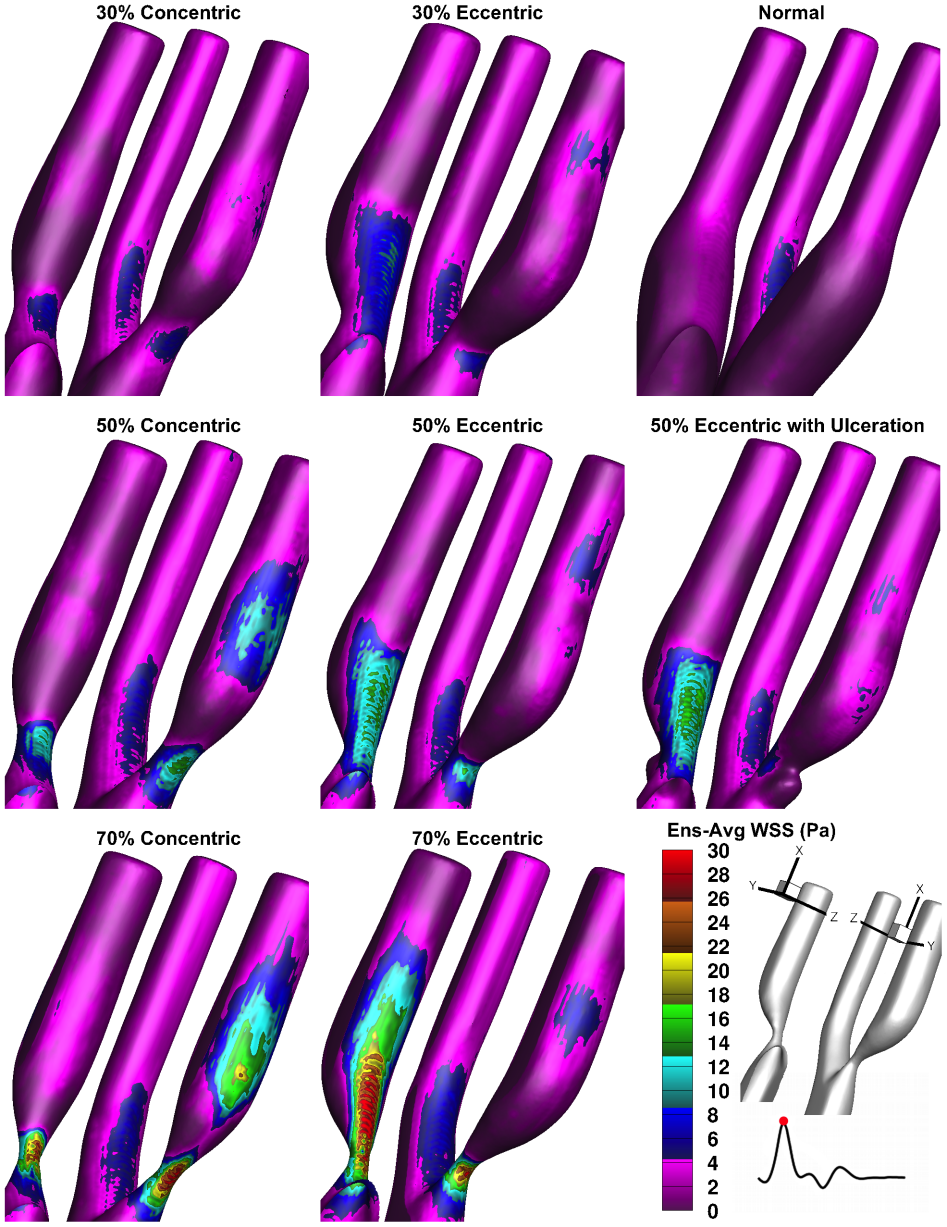
Ensemble-averaged wall shear stress shown for peak systole in the eight carotid models. For optimal display, the ICA is shown from two different perspectives as indicated by the orientation schematic in the bottom right corner. The color bar represents all the models including the normal geometry. For improved accommodation, the dynamic range of the color bar is reduced to a maximum of 30%-stenosed models.

Figure 6 illustrates laminar WSS at the time point of maximum shear stress (i.e. peak systole), showing the models from two viewing angles in order to visualize both the inner and outer ICA walls. The normal model exhibits very low WSS ranging from 0 Pa, (i.e. stagnant fluid) at the outer wall of the ICA bulb and less than 4 Pa along the inner ICA wall, up to a maximum of 6.5 Pa arising at the bend of the inner ECA wall; similar values were consistent in the ECA of all the examined models. Overall, the maximum-WSS ranges were found to be similar in each pair of models with equal stenosis severity, reaching a peak value of 8–10 Pa in the 30% models, 18 Pa in 50% models (non-ulcerated), and 30–36 Pa in the 70% models. Consistently, in all the concentric models, elevated WSS appears at two disjointed regions: within the stenosis throat and distally at the outer ICA wall where the high-velocity jet impinges. In the eccentric geometries (including ulcerated), an elevated-WSS region forms within the stenosis throat and continues distally along the inner wall, along with two regions of slightly elevated WSS on the downstream outer wall due to the jet impingement and the reversed flow that creates the outer recirculation zone.

**Figure 7:**
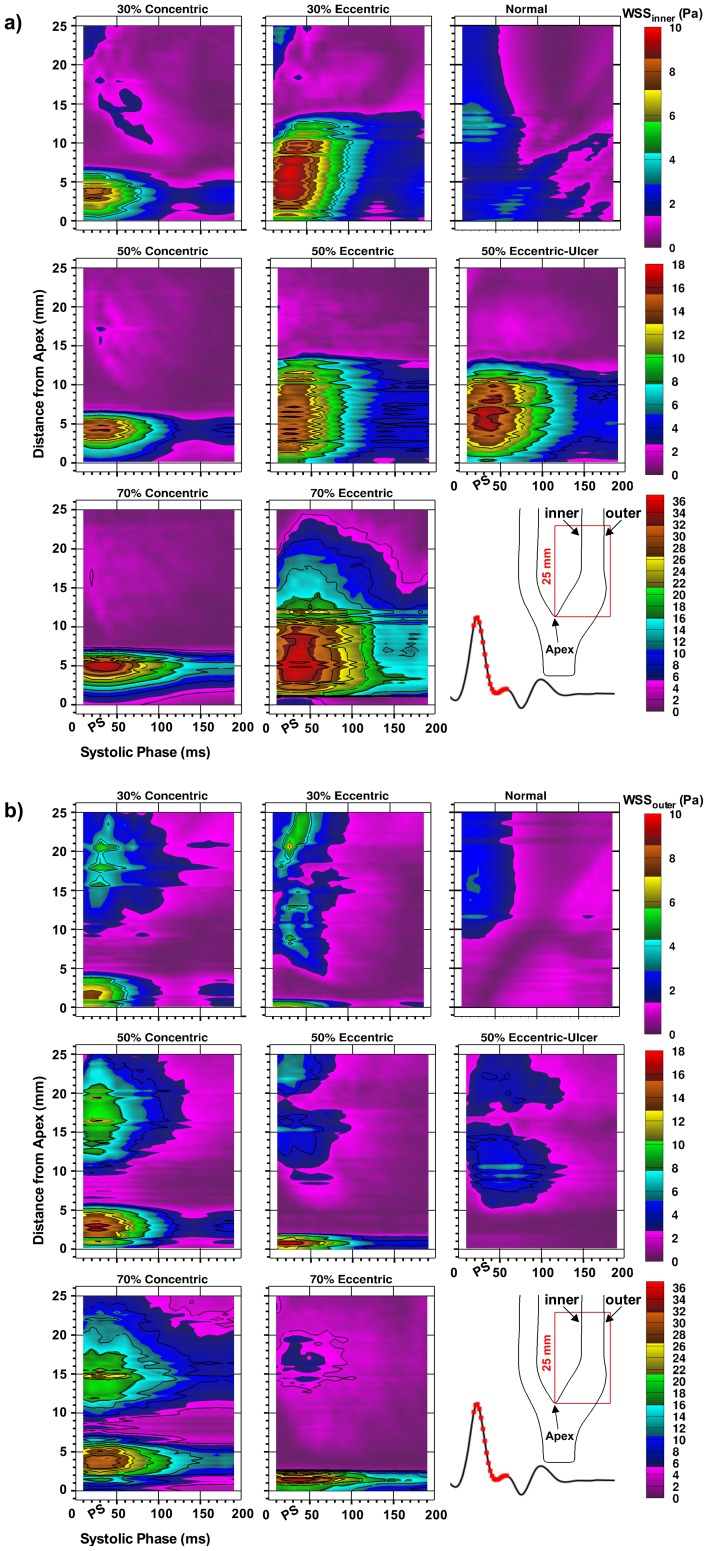
Spatial and temporal distribution of the wall shear stress along (a) the inner wall and (b) the outer wall in the eight carotid models. For each time point, wall-bounded values were extracted along the 25-mm analyzed length starting at the bifurcation apex. The analyzed 180-ms window (with peak systole denoted as PS) is highlighted on the reference flow waveform. Note the range-appropriate color bar associated with each row of models and the 4-Pa contour increments used for all maps.

Figure 7 illustrates both the spatial and temporal distribution of WSS along the inner (flow-divider wall) (Fig. 7a) and outer wall (Fig. 7b) derived for the same region of the ICA and same window of the cardiac cycle as used for FSS in Fig. 5. In the concentric models, elevated WSS within the stenosis throat (i.e. ∼0–5 mm from bifurcation apex) appears equally along the inner (Fig 7a, left column) and outer wall (Fig 7b, left column). Values do not exceed 10 Pa in the 30%-stenosed models, but increasingly extend from 4–5 mm for up to 100 ms in the 50%-concentric model to a 7 mm region lasting over 180 ms in the 70% concentric at the 10-Pa level. In each eccentric model, the extent of the inner-wall WSS elevation is about twice the extension in the counterpart concentric models. At the 10-Pa level, values extend approximately 7 mm for up to 50 ms in the 30%-stenosed models, increasing to 13 mm for up to 100 ms in the 50%-concentric model, and a 20 mm region lasting beyond the 180-ms window in the 70% concentric.

In the ulcerated model, the WSS region associated with the recirculation zone is larger and sustained longer compared to the non-ulcerated counterpart, but WSS levels do not exceed 8 Pa. Similar to FSS, both inner- and outer-wall WSS levels (Fig. 7 a and b) are sustained ∼20 ms longer for the ulcerated model compared to its non-ulcerated counterpart.

**Figure 8:**
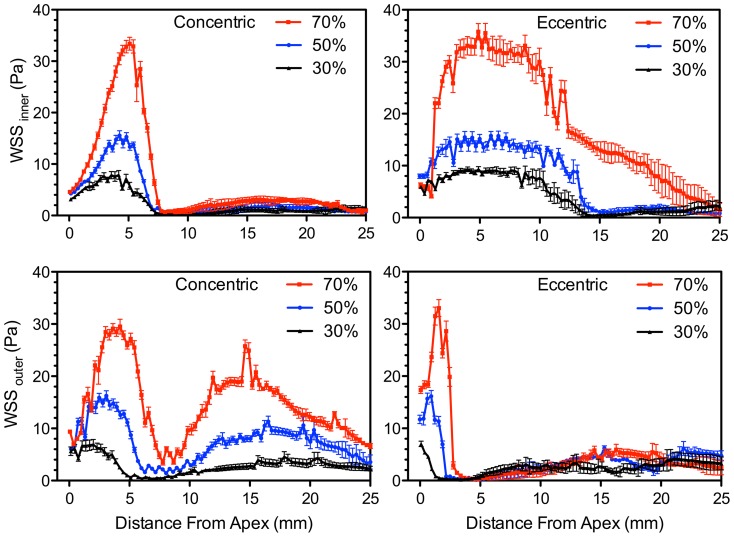
Spatial distribution of inner WSS (upper row) and outer WSS (lower row) associated with an interval during peak systole. Values are mean and standard deviation (whiskers) over five WSS values covering five time points straddling (±20 ms) peak systole.

Of particular interest is the slope of decay from the maximum WSS at the apex of the stenosis, as previous studies have shown the association of this decay gradient with the growth of unstable thrombus immediately downstream of the stenosis [11]. Rates of decay were quantified from Fig. 8, which shows the values of WSS extracted from a 25-mm wall length starting at the bifurcation apex. For both groups of plaque symmetry, the maximum WSS and subsequent decay from the maximum WSS (along both inner and outer wall) increases with increasing stenosis severity. For the outer-wall WSS, the slope of decay in the concentric models with 30%, 50%, and 70% stenosis were approximately −2, −6, and −9 Pa/mm respectively. The slope of decay in the eccentric models was significantly higher with decays of −5, −12, and −33 in the 30%, 50%, and 70% stenosis respectively, corresponding to increases of 150%, 100%, and 270% over their concentric counterparts.

While the spatial shear gradient in the study by Nesbitt et al. [11] was significantly higher, as it corresponds to a micro-scale spatial zone, spatial shear gradients have also been implicated in previous studies with regional correlation to endothelial cell morphology and the attachment of leukocytes in regions with high spatial WSS gradients over a scale comparable to carotid stenosis [44].

### Reynolds shear stress (RSS)

**Figure 9:**
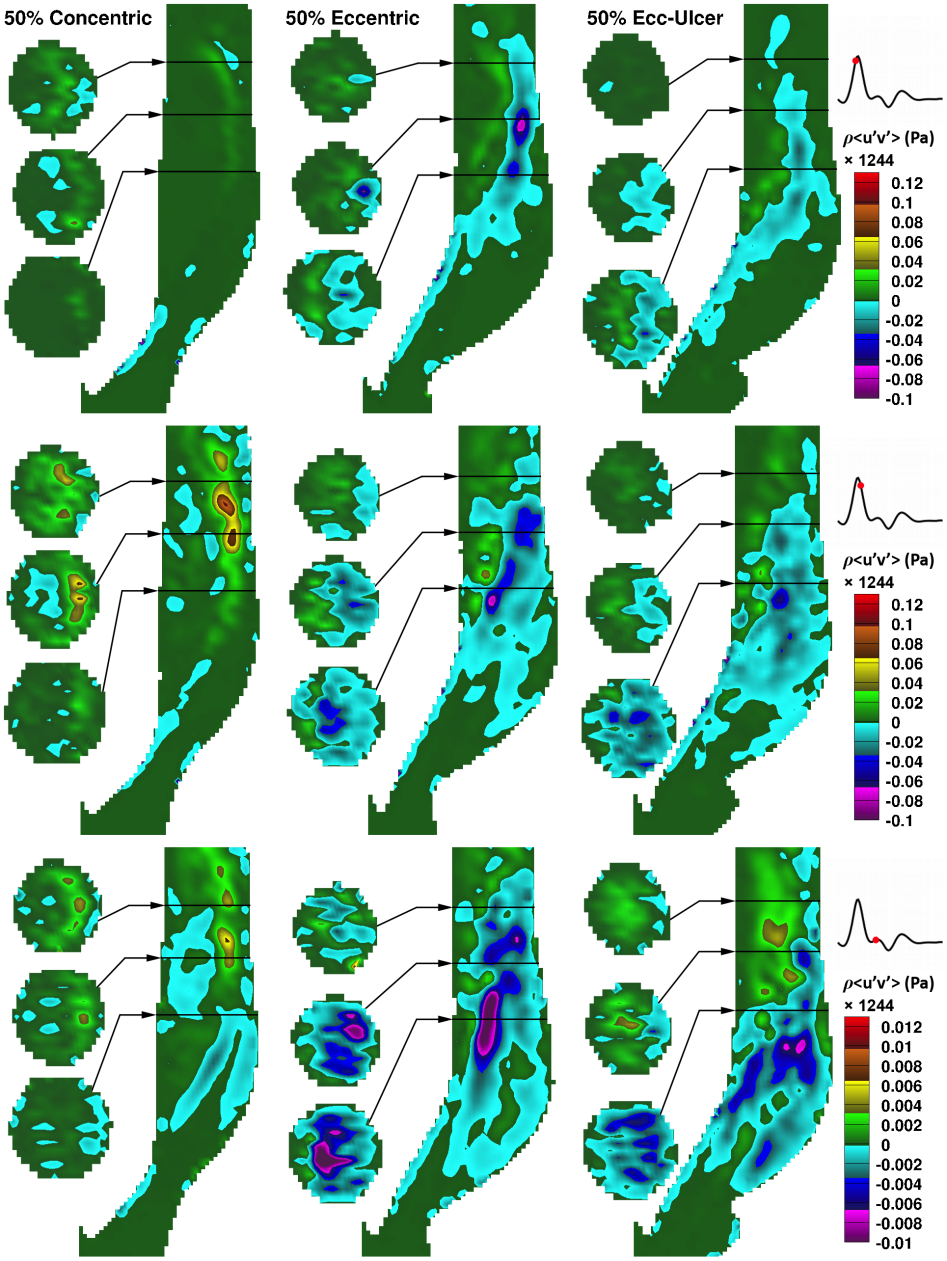
In-plane RSS component (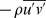) as a function of time for the three 50%-stenosed models. Maps are shown at three time points: 20 ms before peak systole (top row), 20 ms after peak systole (middle), and 20 ms before the dicrotic notch (bottom), as indicated by the respective flow-rate waveforms on the right. Three cross-sectional slices are shown alongside the ICA lumen at the locations indicated by the black lines at approximately 2, 2.5, and 3 CCA diameters downstream (i.e. ∼16, 20, 24 mm). Note the separate color bar used for the bottom row with the same zero-value color as for the upper rows.

Figure 9 shows the distribution in the RSS component of (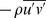) in the 50%-stenosed models at three different time points of the systolic phase: approaching peak systole (systolic acceleration), post peak systole (early deceleration), and near the dicrotic notch (late systolic phase). Prior to peak systole, RSS is concentrated along the jet path. In the concentric model, RSS is skewed toward the outer wall, whereas in the two eccentric models, it is more uniformly distributed over the ICA lumen. During deceleration in the eccentric (both non-ulcerated and ulcerated) models, RSS distribution expands into the large outer-wall recirculation zone reaching a maximum RSS (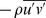) value of about 25 Pa. The RSS distribution in the large recirculation zone persists during the entire deceleration phase, as evident by the third time point shown, although covering a lower range (∼5 Pa maximum RSS). In the concentric model, spotted regions of elevated RSS are observed downstream during the deceleration phase, unlike the large distribution seen in the eccentric counterparts.

**Figure 10:**
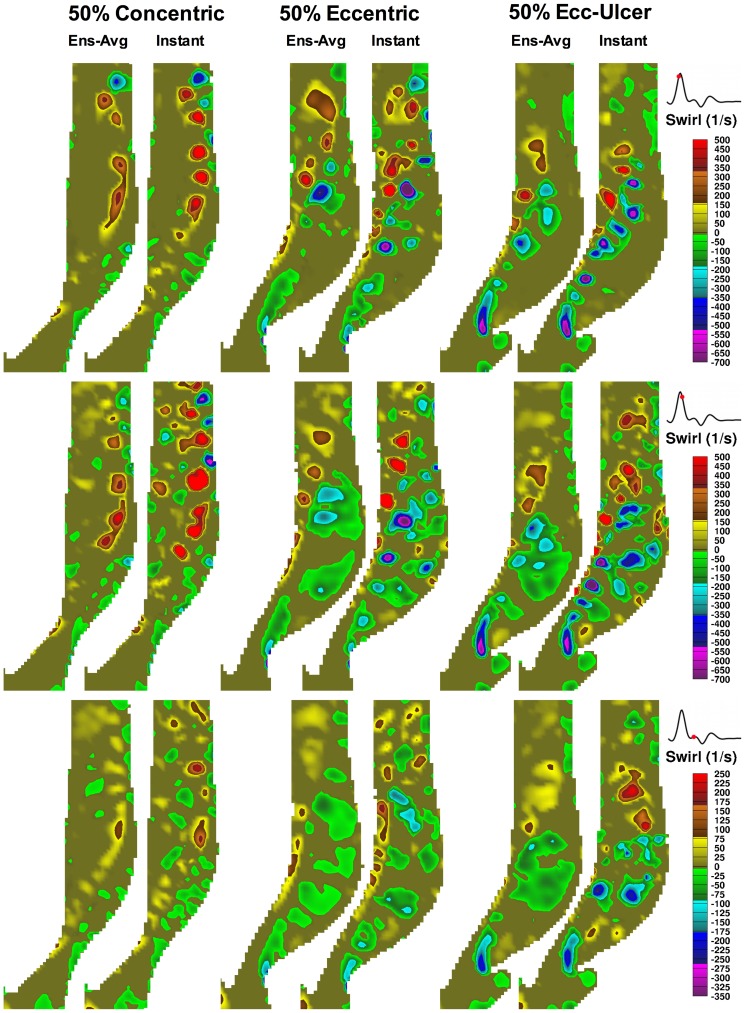
Color-encoded maps of orientation-dependent swirling strength (λ_ci_) shown for the ICA central plane in each of the set of three 50%-stenosed models at time points identical to those applied to Fig. 9. Swirling strength maps were obtained from x and y components of the ensemble-averaged (left panel in each column pair) and instantaneous (right panel in each column pair) velocity maps. Note the different limits of the color bar used for the bottom row. Positive (e.g. yellow to red) and negative values (e.g. green to violet) represent counter clock-wise and clock-wise swirling rotation, respectively.

As expected, RSS is governed by flow instabilities and coincides with the presence of vortices. Figure 10 shows central-plane vortices identified using swirling strength [39] in the three 50%-stenosed models derived from either an ensemble-averaged velocity map (left panel of each pair) or an instantaneous velocity map (right panel of each pair) for the same three cardiac phases shown in Fig. 9. During the acceleration phase (top row), flow is well organized, with vortex shedding appearing along each side of the jet in all three models. Due to increasing flow instabilities during systolic deceleration (middle row), vortices also form in the post-stenotic region, particularly as the large outer recirculation zone breaks down into small vortices in the eccentric models. Destabilization of the helical flow in the concentric model can also be seen as small instabilities in the jet-free region. At the late systolic phase (bottom row), the coherent vortex shedding along the jet disappears, but flow instabilities are still present in the recirculation zones.

**Figure 11:**
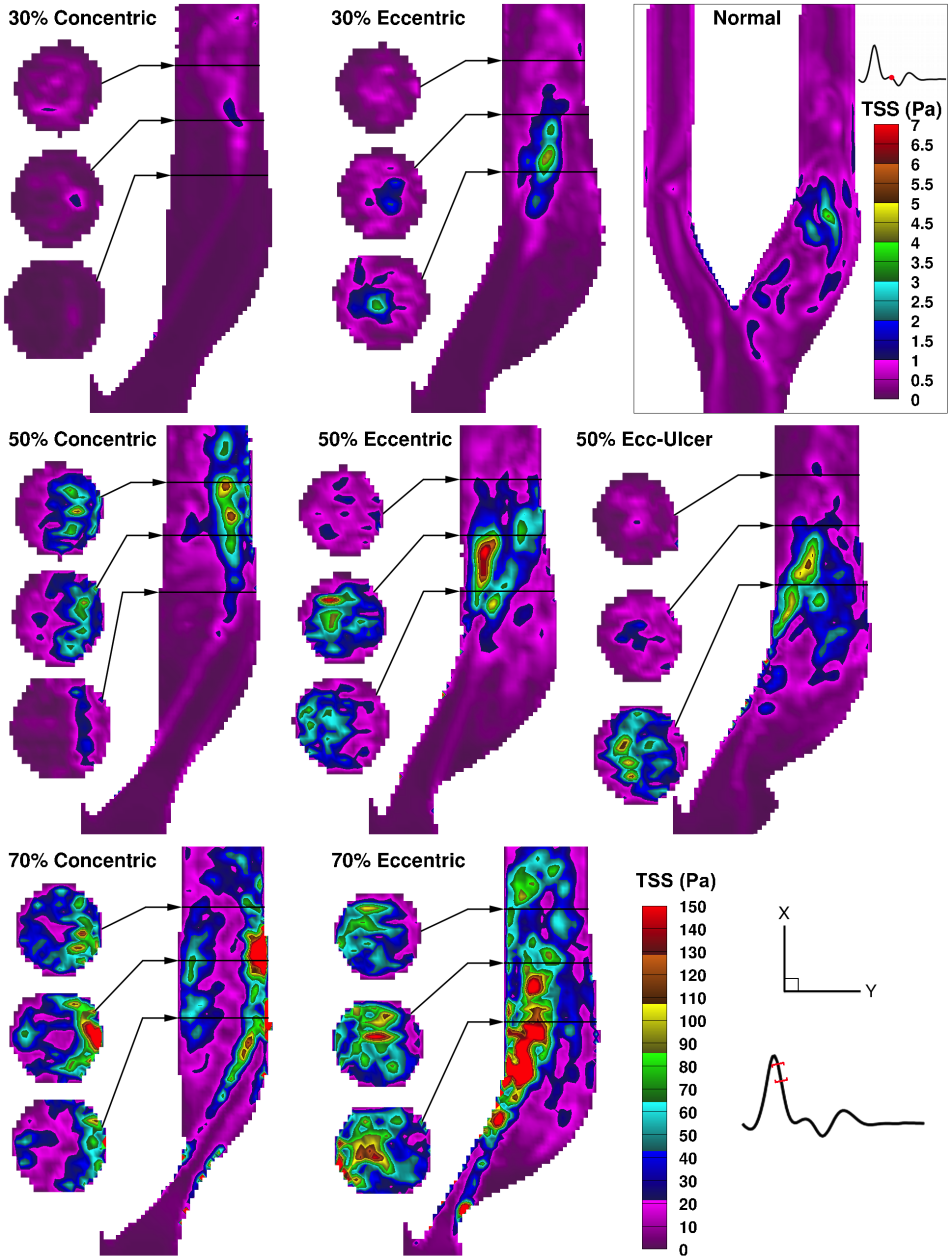
Color-encoded maps of total shear stress for the time point of maximum TSS in each of the eight carotid models. For each model, a map demonstrating maximum TSS is shown, where the time point corresponding to the maximum value varied only slightly (20±10 ms beyond peak systole) for the eight models. Note the bottom right color bar (set to the maximum of 150 Pa) represents the seven stenosed models; the time and color bar associated with the normal model are shown in the enclosed box.

Figure 11 shows total shear stress (TSS) maps at the time point of maximum value for each model, which occurs early within systolic deceleration for all the examined models, with the exception of the normal model. The elevated TSS regions are primarily contributed by elevated RSS, while minor contributions from FSS (Fig. 5) appear in lower values along the jet. In the 30% concentric model, TSS seems to follow the FSS pattern due to a minimal level of flow instabilities. The elevated region of TSS expands starting from the point of jet impingement located at the post-stenotic outer wall in the concentric models and from the downstream detachment point located at the inner wall in the eccentric models. Overall, the maximum TSS value increases rapidly with increasing stenosis severity as seen in the concentric models, with maximum TSS values of 32, 122, and 298 Pa for 30%, 50%, and 70% stenosis severity respectively, and similarly in the eccentric models, with values of 99, 158, and 303 Pa. Notable from the cross-sectional slices, the elevated-TSS volume is mainly bounded to the outer wall in the concentric models, and more fully distributed in the eccentric models due to the larger distribution of flow instabilities when the tail of the jet crosses from the inner to outer wall. These patterns of elevated TSS also correspond to elevated turbulence intensity distributions previously illustrated using PIV [40].

**Figure 12:**
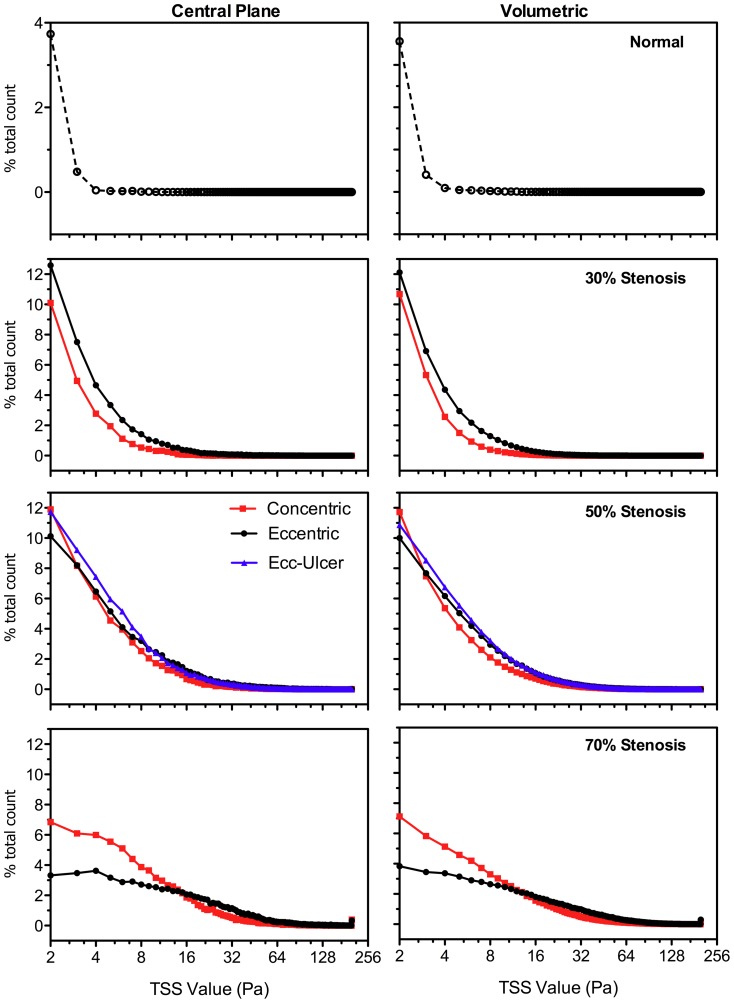
Histogram of normalized distribution of TSS (≥2 Pa) for the ICA branch (0–35 mm distal to bifurcation apex) and over the systolic phase (same 180 ms window depicted in Fig. 5). Plots represent the number of voxels accumulated over time and space for each TSS value (1-Pa bins) normalized by the total number of non-zero voxels accumulated for the same time frame in each model. Central plane (left column) includes ∼2000–2500 voxels and volumetric data (right column) includes ∼21000–32000 non-zero voxels, depending on geometry. Note, horizontal axes are presented in log-2 form, all values exceeding 200 Pa fall within the 200-Pa bin, and the normal model uses a different range for the vertical axis.

Figure 12 demonstrates the relative distribution of TSS values (i.e. combined laminar and turbulent shear stress) for spatial and temporal accumulation over a systolic 180-ms time window (as depicted in Fig. 5) for a downstream region extending laterally wall-to-wall and 35 mm distal to the apex in the ICA branch. Distributions are compared for the central plane alone and for the volumetric data (based on 15 planes). In the 30% stenosed models, higher levels are seen across all TSS values in the eccentric model. In the 50% and, more pronouncedly, in the 70% eccentrically stenosed models, this increase was found for higher-level shear stress (i.e. elevated wall and turbulent shear stress) as observed for values above 15 Pa in the 70% models. The ulcerated and non-ulcerated 50%-stenosed models were similar, in general, with greater accumulation found for low-level shear stress (below 8 Pa) in the ulcerated model, as similarly shown for low levels of turbulence intensity based on PIV [40].

## Discussion

We have provided a detailed analysis of shear-stress spatial distribution and time evolution for varying carotid plaque geometries. Two factors were observed that can potentially contribute to a higher level of thrombosis due to increasing stenosis severity: 1) increased shear-stress levels (both free and wall) for each examined time point (Figs 5 and 7) and 2) a steeper slope of decay descending from the maximum shear stress at the stenosis apex (Fig. 8).

While the effects associated with increasing stenosis severity can intuitively be expected, our findings also demonstrated marked differences between concentric and eccentric plaque symmetry. In the eccentric models, four notable issues arise, compared to the concentric symmetry, that may contribute to increased risk of thrombosis: 1) significantly extended WSS formed along the inner wall (Fig. 7a); 2) significantly steeper decay of WSS just beyond the stenosis apex along the outer wall at the leading edge of the large recirculation zone, which has been shown in vivo to contribute to larger growth of unstable thrombus [11] (Fig. 7b); 3) elevated and dispersed levels of RSS in the post-stenotic recirculation zone (Fig. 9); and 4) a larger expansion of turbulent shear stress associated with jet instabilities (Fig. 9 and 11).

The high level of intraluminal total shear stress in all the stenosed models, which increased ten-fold (from ∼30 Pa to 300 Pa) with increasing stenosis severity, is suggestive of increased potential for thrombosis. Reports by Holmes et al. [45] showed that a shear-stress level of at least 30 Pa (corresponding to shear rate of 10500 s^−1^), when present even for a very short time (0.075 s), resulted in platelet activation and formation of platelet micro-particles. Additionally, our results showed that plaque eccentricity resulted in either higher levels or larger expanses (or both) of turbulent shear stress compared to concentric geometry, thus also suggesting potential for increased thrombosis activity.

Plaque ulceration has shown association with the presence of thrombus [46]. Based on our findings in an ulcerated 50% eccentric model, ulceration potentially contributes to a higher level of thrombosis activity due to: the longer sustention (∼20 ms) of elevated FSS and WSS (Fig. 5 and 7) during systole, larger expansion of RSS due to a larger recirculation zone (Fig. 3), and the adjacency of the shear stress lamina to the low circulating flow of the ulcer crater.

We introduced a new metric of assessing shear stress separately via laminar and unsteady conditions. The ensemble-averaged (referred to here as laminar) shear stress represents the dominant levels of shear stress under which the thrombotic activity occurs. The high-level turbulent shear stress, in which cycle-to-cycle variability is included, occurs less often, as was shown in Fig 12. Using instantaneous peak WSS can be a misleading metric since the cycle-to-cycle variability is not taken into account – potentially explaining the variation in peak WSS found between studies.

Moreover, in the literature, almost no attention is given to intraluminal free shear stress as evaluated in the present study. Free shear layers are generated at the interface of the jet and separated flow regions (e.g. recirculation zones); during the systolic phase, when this sheared interface became unstable, vortex shedding appeared along the sides of the shear layers, as seen in Fig. 10. The significance of vortex shedding in the thrombosis mechanism has been demonstrated previously [47], where particle paths showed that platelets, after being exposed to high-level shear stress layers, were entrapped within the vortices shed beyond heart-valve leaflets.

Besides shear stress, certain flow patterns can also play a significant role in thrombosis; for example, the presence of recirculation results in longer residence times and thus higher platelet activation level [25]. During the systolic deceleration phase, when flow becomes most disturbed [27], [39], [48], additional vortices appear due to the break down of the large recirculation zone (Fig. 9) that was prominent in the eccentric models. It has been shown [49] that these post-stenotic vortices can enhance the local deposition and convection of the platelets near the wall (i.e. potentially near the site of plaque rupture). This region also was associated with the presence of elevated RSS during the deceleration phase (Fig. 9), and thus of potential significance for platelet activation and RBC hemolysis. Turbulent flow conditions promote high pathological shear-stress levels, thus suggesting that shorter exposure times should be required for platelet activation. Although platelets can survive damage from extremely short exposure time (in the order of 10^−5^ s) [19], it has been shown that a high level of shear stress can sensitize the platelets and prime them for activation under steadier flow conditions [50]. However, it has also been suggested that thrombosis under turbulent conditions may imply a different mechanism, as platelets would need to form bonds very rapidly [51].

Shear stress is extremely difficult to estimate experimentally. PIV provides spatial and temporal resolution that is sufficiently high to allow adequate estimation of velocity gradients. However, PIV has a known challenge in shear estimation, imposed by the standard cross-correlation algorithm, that results in biasing the velocity estimation near walls (e.g. due to high velocity gradients and lower concentration of particles) [41]. The biased uncertainty in velocity estimation near the wall can be significantly reduced by employing advanced PIV algorithms, such as the one applied here, that include window and image deformation to account for lost particle pairs and velocity gradients [52]. Moreover, the accuracy of PIV velocity estimation can be increased by enhancing the quality of the particle images by applying recommended precautions, as applied here, such as reducing wall reflections by using a well-matched fluid, use of fluorescent particles to significantly improve the image intensity compared to the standard silver-coated particles, and the use of an appropriate optical filter to reduce the unwanted background illumination.

As discussed in the Methods section, the mask applied to separate flow from the non-flow region was an RMS-intensity mask created from a cardiac-cycle series of particle images (created individually for each plane of measurement). The benefit of our RMS mask, as opposed to the commonly used manual masking, is that it specifically eliminates the regions with a constant intensity through out all the images. As a result, the areas outside the lumen and a narrow line along the inner wall, where some particles are stuck to the wall, were eliminated, and only flow regions were kept (Fig. S2). From the inner wall, it is estimated that a *maximum* of 4 pixels have been eliminated along the flow-region boundary. Due to this elimination, as well as elimination of vectors located within half of an interrogation window from the wall-normal, and having a vector spacing of 8 pixels (minimum 16×16 interrogation window with 50% overlap), it is estimated that the effective location of the wall (i.e. true zero-value velocity vector), must lie within 8–16 pixels from the last captured non-zero vector. Therefore the two zeros beside the last valid vector, with 8-pixel vector spacing (corresponding to ∼0.3 mm), were included in the central differencing, and WSS was calculated at the location of the first zero-velocity vector adjacent to the last non-zero vector. It should be noted that this estimation conservatively results in an underestimation of WSS, rather than an overestimation. However, if the effect of the mask in eliminating part of the wall is ignored and only the first zero adjacent to the last non-zero vector accounts for the wall location, then a three-point forward-backward differencing can be applied along the wall. In this case, WSS values will be overestimated and significantly higher compared to the central differencing method (Fig. S3). Thus, in the present work, the central differencing method has been applied to account for the wall location and to avoid overestimating the WSS values. The uncertainty in the estimation of laminar WSS is also related to the size of the sample used for averaging and the level standard deviation (i.e. turbulence). The regions with highest expected WSS uncertainty are thus the regions that contain elevated turbulence coinciding with the distal jet detachment (Fig. S4). It can be observed that for the reported regions of elevated WSS, which includes the stenosis throat and immediate post-stenotic region, the level of expected noise is a minimum.

The presented work is the first PIV-based shear-stress estimations in a comparative set of carotid artery models. Very few experimental carotid shear-stress estimations are reported, with most based on clinical modalities, including phase-contrast magnetic resonance imaging (pcMRI), [53] and echo-PIV (ultrasound-based PIV), which has recently shown potential for *in vivo* full-field velocimetry [54], [55]. The majority of the shear-stress studies in the literature have employed numerical simulation methods in a wide variety of study-specific geometries and including primarily laminar flow models [32], [56], [57] plus a few turbulent models [27], [31], [58]. Depending on the type of the simulation model, inlet flows, and examined geometries, various values of shear stress have been reported; for instance, for a 50% stenosis at peak systole, the two turbulent-model studies by Birchall et al. [31] and Xue et al. [58] reported a peak instantaneous WSS of 120 Pa and 180 Pa, respectively. This can be considered consistent with our findings of TSS rising up to 130 Pa in the wall of the 50%-stenosis throat. Employing a laminar flow model, Schirmer and Malek [59] reported a peak WSS of 87 Pa at the throat of an axisymmetric 50%-stenosed model. For a 75% stenosis (based on NASCET criteria), however, their reported value reached 424 Pa, which is in close proximity to the value reported by Birchall et al. for a 90% stenosis (also based on NASCET criteria).

The PIV-measured WSS results in this study matched well qualitatively with CFD-predicted results shown previously by Steinman et al. [32] in the identical 30% models, despite a twofold higher *peak WSS* compared to our PIV results (20 Pa vs. 10 Pa, respectively). Besides differences in flow rates, fluid viscosity, and effective lumen geometries, this discrepancy can be attributed to the fact that PIV-measured WSS, as discussed earlier, may be underestimated due to uncertainty in the exact location of the wall. However, it is important to note that even when using closely matched PIV experimental parameters for CFD input conditions, previous CFD-PIV comparison studies have reported discrepancy between the two methods, as seen in two CFD-PIV aneurysm studies by Ford et al. [60] and Raschi et al. [61]. Both studies showed matched flow patterns between CFD and PIV, but interestingly both also reported higher near-wall velocities predicted by CFD compared to PIV measurements, which translates to higher CFD-based WSS.

The emphasis of our study was to investigate the comparative patterns and levels of shear stress within carotid models with varying plaque geometries. In order to investigate the direct association between shear stress and the resulting level of thrombosis activity, further understanding of platelet interactions within flow dynamics is required, which has inspired trajectory-based studies [25], [47], [62]–[65] with many interesting reported findings. For instance, the numerical study by Massai et al. [65] showed that the presence of helical flow in the stenosed ICA results in moderation of shear-induced activation for the transported platelets, with a larger helical flow index being associated with less activation of platelets.

As mentioned earlier, thrombosis at the plaque site can be induced by high pathological shear stress in different ways, such as by promoting plaque rupture, thus exposing the lipid core, or by spatially regulating superimposed thrombus on the plaque surface. Thus, the differences in shear stress levels and patterns reported in the present work may explain how certain geometrical features of the plaque can be correlated with more frequent cerebrovascular events. However, it is important to note that, besides altering the local hemodynamics, geometrical features of the plaque are interlinked with plaque composition; eccentric plaques have been reported to be strongly associated with higher content of lipid [66], and plaque composition changes to more soft and haemorrhagic plaque with increasing stenosis severity [67]. These findings suggest that a combination of physiological and hemodynamic factors contribute to an increased risk of cerebrovascular events. Albeit this may be via an inherent feedback mechanism where geometry effects hemodynamics, which effect plaque build up and evolution, thus further changing geometry and hemodynamics.

Potential limitations of the present work lie in the idealized models and Newtonian blood-mimicking fluid implemented here. The use of a Newtonian blood-mimicking fluid can be considered valid under the well-established and common assumption of Newtonian behavior of blood in large arteries [68]. The idealized carotid phantoms do not include vessel tortuosity or physiological compliance and can be considered essentially rigid. Velocity profiles, and consequently the shear stress patterns, can also be impacted by the vessel compliance and wave reflection [69], as compliance is known to provide a buffering capacity to provide sufficient steady blood supply throughout the cardiac cycle. Several matched-model studies have compared between a rigid and compliant model demonstrating flow differences associated with the compliance, although typically in the context of early development of atherosclerosis in healthy (non-stenosed) geometries. In a numerical study with fluid-structure interaction in a normal carotid artery, a 25% decrease in WSS was seen in the compliant model compared to rigid [70], however, the global structure in the flow and shear patterns were unchanged. Reflection of a pressure wave generally occurs due to impedance mismatch attributed to geometrical variation in the arterial tree, such as branching or narrowing of the lumen, as well as due to the reduced arterial compliance. Besides the impact of altering the flow-rate amplitude, wave reflection results in phase angle variations [71] where the level of WSS has been shown to be sensitive to the phase relations between pressure, flow rate, diameter, and WSS, which were altered due to wave reflections that in turn were mediated by the magnitude of wall motion in an elastic tube. However, an advantage of the current matched-model study is that it allows us to independently studying the effects of stenosis severity, plaque eccentricity, and ulceration, before introducing the additional variable of compliance. Our work in progress includes investigating the impact of compliance through matched-model studies in identical diseased carotid geometries incorporated in compliant phantoms.

While the significance of stenosis severity is undeniable, alone and (especially for moderate cases), it is not a sensitive predictor of stroke risk [72]. In the quest for more sensitive risk assessments, additional factors that increase the possibility of thrombosis, such as plaque vulnerability, have been suggested for consideration as stroke-risk factors, [73]. The notable differences in shear-stress levels and patterns found in the present work, as well as the marked differences in flow-disturbance levels previously reported [40], would reasonably be expected to translate to different levels of thrombosis activity, thus suggesting the potential diagnostic benefit of considering plaque eccentricity and ulceration, in addition to the stenosis severity. With the experimental knowledge and validation base provided here for the first time in a comprehensive series of carotid models, clinical shear-stress estimations, which have shown promising results [74], and further trajectory-based studies are encouraged for enabling clinically relevant estimation of the thrombosis risk levels based on geometrical features of the stenosis.

## Supporting Information

Figure S1 **Velocity profiles (a) across that CCA at 2 CCA diameters (16 mm) upstream of the bifurcation apex in the normal carotid artery model for the eight time points (with corresponding colors) indicated on the flow-rate waveform (b).**
(TIFF)

Figure S2 **Example of an unmasked particle image subtracted from the corresponding masked image.** The inner area with zero intensity (black) is the area that has been included in evaluation of valid velocity vectors.
(JPG)

Figure S3 **Comparison of shear stress in the 50% concentric model as calculated from the two differencing schemes: a) forward differencing and b) central differencing; the more conservative central differencing scheme was used elsewhere for all other figures.**
(TIF)

Figure S4
**Expected regions of elevated WSS uncertainty.** For each component of mean velocity (such as Ū), the measurement noise is estimated by 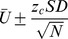, which for our measurements, the confidence coefficient (*z_c_* = 2.145), is determined from the t-distribution table for a sample size of N = 15. For each point, the laminar strain tensor has been calculated once with the maximum range of mean velocities (calculated as explained above) and once with the minimum range of velocities; the difference between these corresponding WSS values (b) is shown compared to the WSS based on the reported mean values (a).(TIF)

Movie S1 **Comparison of ensemble-averaged velocity magnitude over the cardiac cycle for the eight examined carotid artery bifurcation models.** For optimal visualization of the broad range of velocities across the models, a common color bar is used with the maximum representing all velocities ≥2 m/s; the actual maximums reach 3.2 and 3.4 m/s in the 70% concentric and eccentric models, respectively, as seen in Fig. 3.
(MP4)
