# Supplementary figures and images for: In Vitro Shear Stress Measurements Using Particle Image Velocimetry in a Family of Carotid Artery Models: Effect of Stenosis Severity, Plaque Eccentricity, and Ulceration
Source: PLoS One. 2014 Jul 9;9(7):e98209. doi: 10.1371/journal.pone.0098209 (PMC4090132; doi:10.1371/journal.pone.0098209)

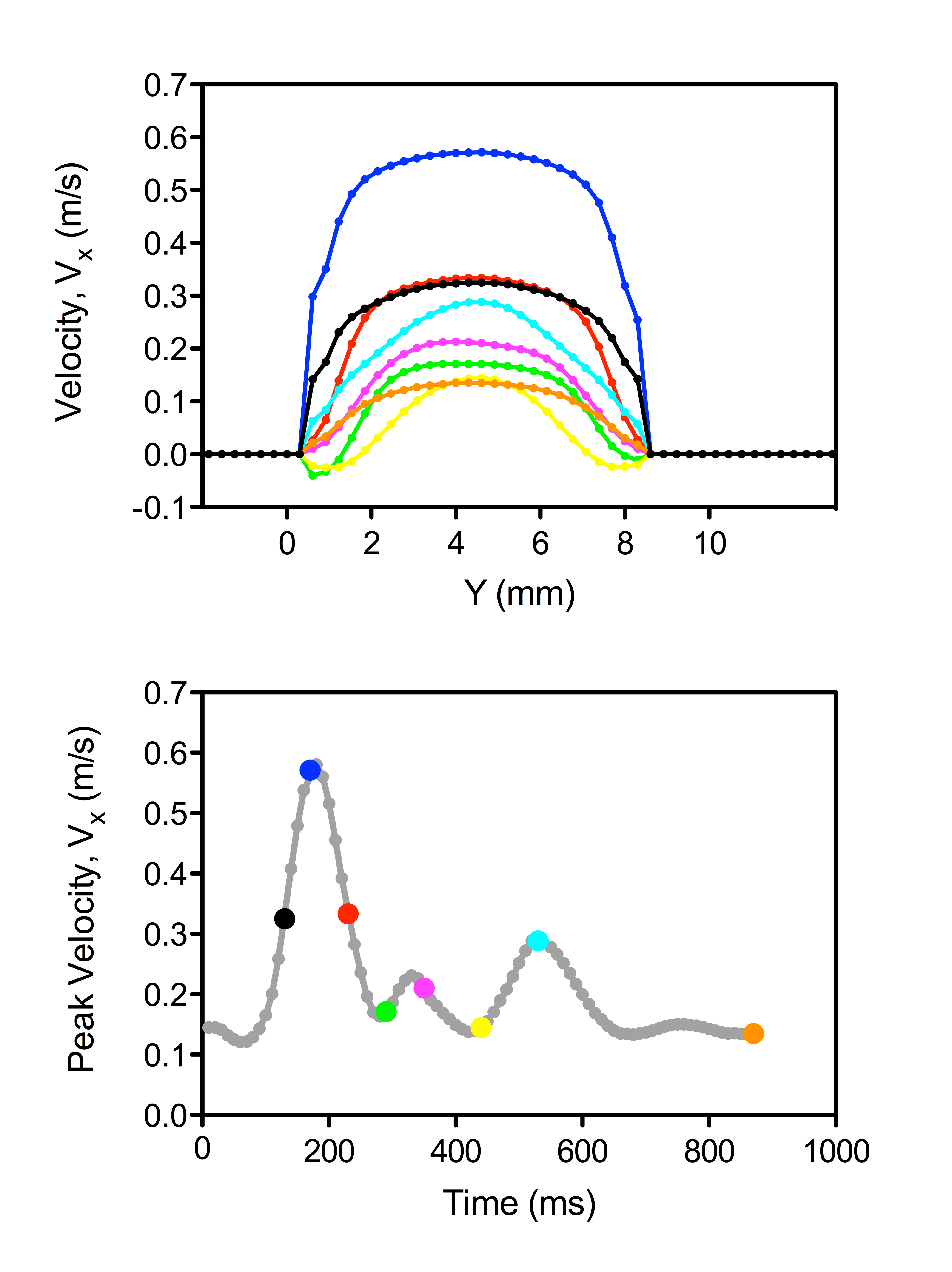

Supplement: Figure S1 — Velocity profiles (a) across that CCA at 2 CCA diameters (16 mm) upstream of the bifurcation apex in the normal carotid artery model for the eight time points (with corresponding colors) indicated on the flow-rate waveform (b). (TIFF) [file pone.0098209.s001.tiff]

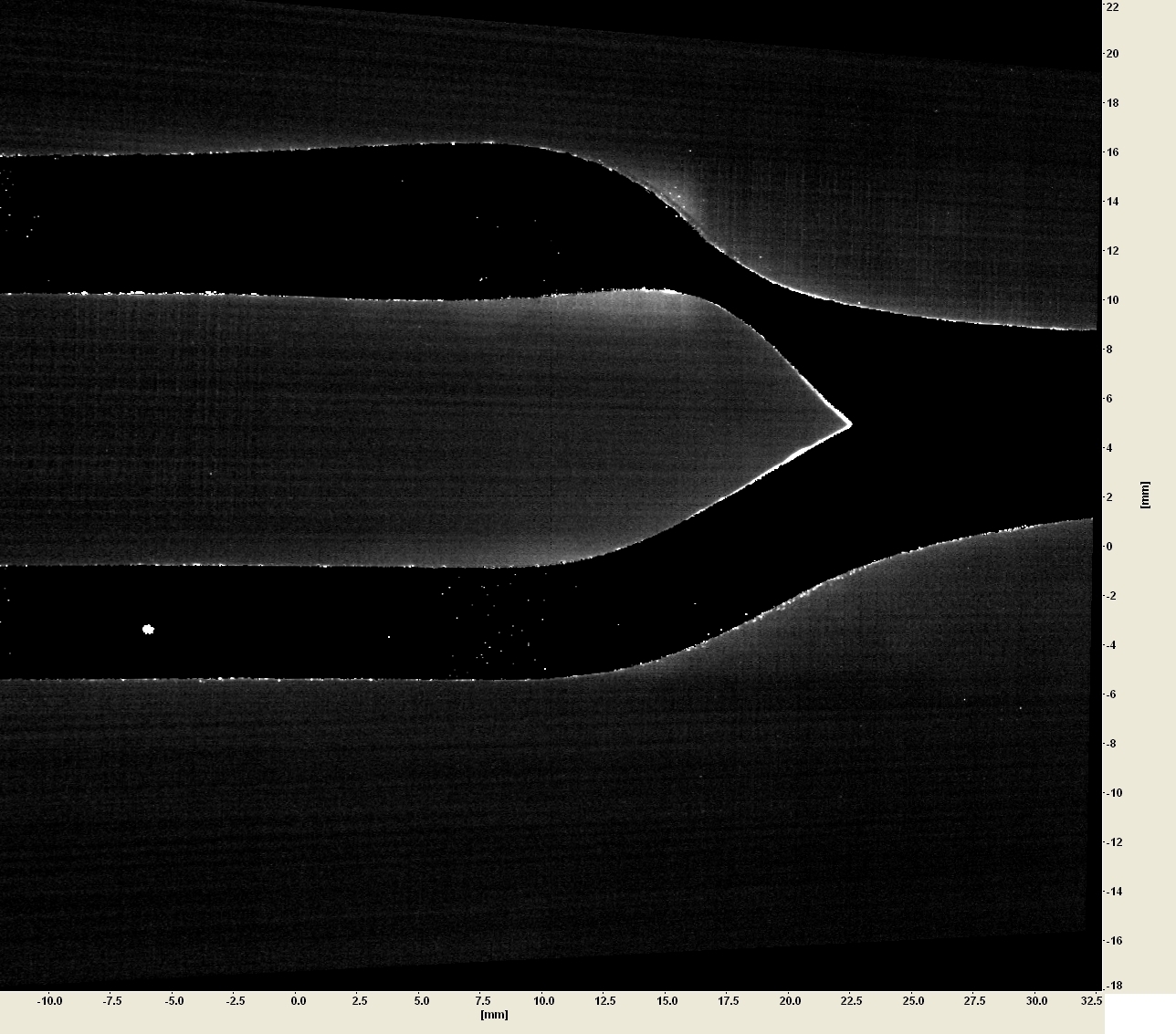

Supplement: Figure S2 — Example of an unmasked particle image subtracted from the corresponding masked image. The inner area with zero intensity (black) is the area that has been included in evaluation of valid velocity vectors. (JPG) [file pone.0098209.s002.jpg]

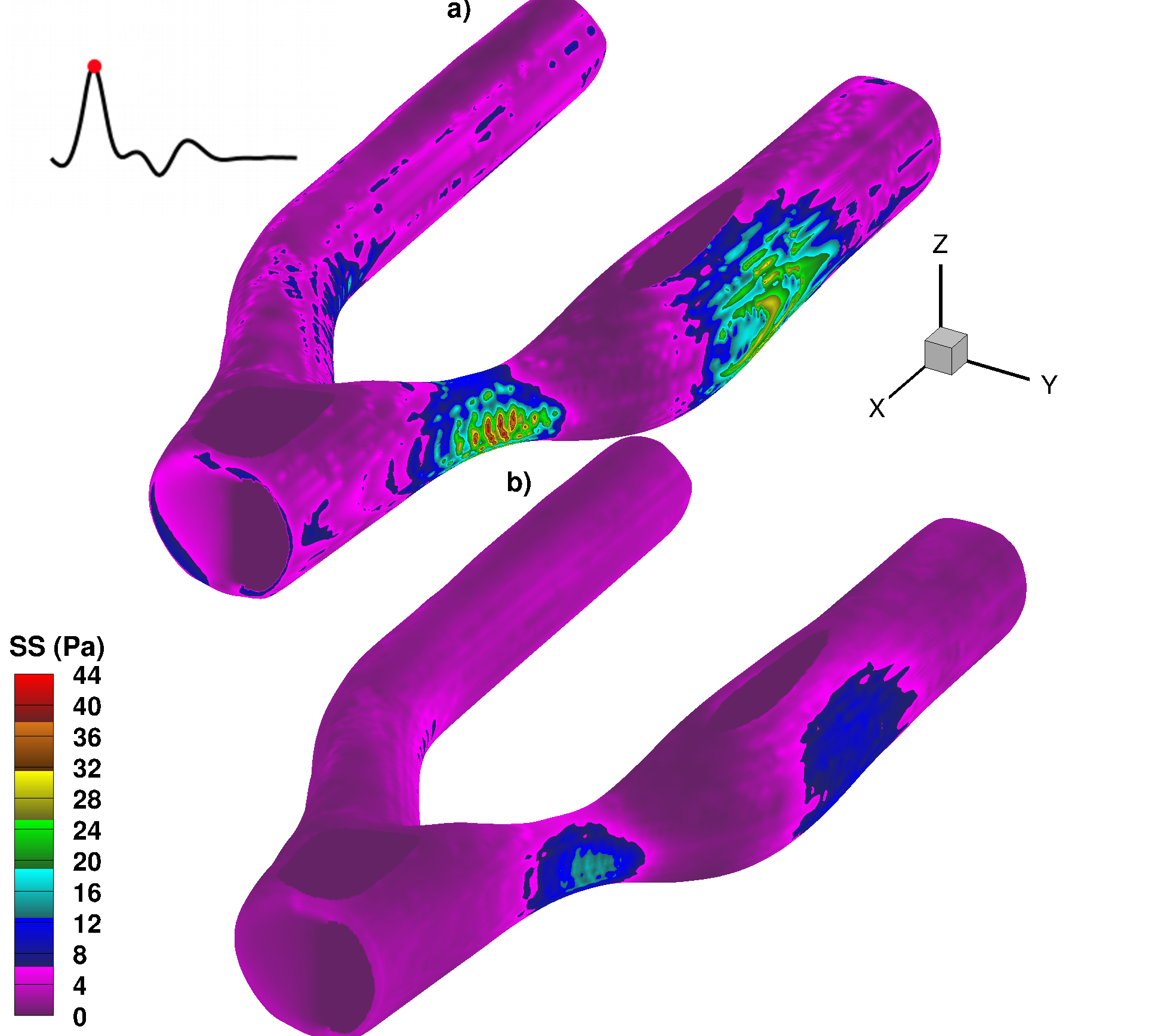

Supplement: Figure S3 — Comparison of shear stress in the 50% concentric model as calculated from the two differencing schemes: a) forward differencing and b) central differencing; the more conservative central differencing scheme was used elsewhere for all other figures. (TIF) [file pone.0098209.s003.tif]

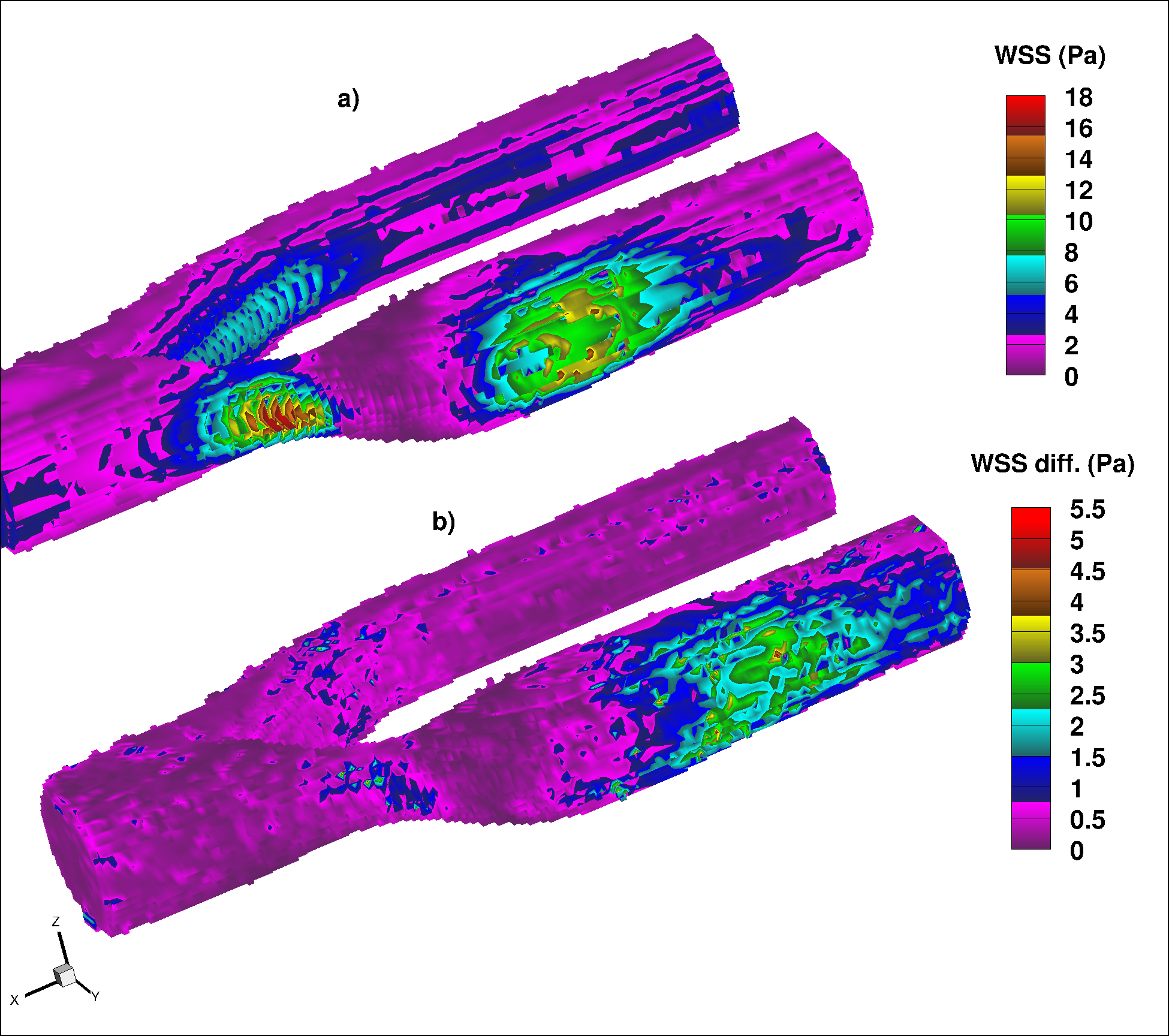

Supplement: Figure S4 — Expected regions of elevated WSS uncertainty. For each component of mean velocity (such as Ū), the measurement noise is estimated by , which for our measurements, the confidence coefficient (zc = 2.145), is determined from the t-distribution table for a sample size of N = 15. For each point, the laminar strain tensor has been calculated once with the maximum range of mean velocities (calculated as explained above) and once with the minimum range of velocities; the difference between these corresponding WSS values (b) is shown compared to the WSS based on the reported mean values (a). (TIF) [file pone.0098209.s004.tif]
